# Maternal weight gain and intestinal permeability marker zonulin in late pregnancy: implications for nutritional and metabolic health

**DOI:** 10.1186/s40795-026-01292-6

**Published:** 2026-02-27

**Authors:** Tuğba Küçükkasap, Elif Rana Akbey, Aziz Kından, Yıldız Akdaş Reis, Yaprak Engin Üstün

**Affiliations:** 1 Department of Nutrition and Dietetics, Health Sciences University, Ankara, Turkey; 2 Department of Nutrition and Dietetics, Institute of Health Sciences, Hacettepe University, Ankara, Turkey; 3 Department of Perinatology, Aziz Kından, Etlik City Hospital, Ankara, Turkey; 4 Department of Obstetrics and Gynecology, Etlik City Hospital, Ankara, Turkey; 5 Ankara City Health Application and Research Center, Health Sciences University, Ankara, Turkey

**Keywords:** Maternal nutrition, Maternal obesity, Zonulin, Intestinal permeability, Oxidative stress, Fetal growth

## Abstract

**Background:**

Gestational weight gain (GWG) outside recommended ranges has been associated with adverse maternal and neonatal outcomes; however, the underlying biological mechanisms remain incompletely understood. Intestinal permeability, assessed using serum zonulin, has emerged as a potential pathway linking metabolic and inflammatory changes during pregnancy.

**Methods:**

This cross-sectional study included 51 pregnant women with normal pre-pregnancy body mass index and their neonates. Participants were classified according to gestational weight gain as within or above recommended limits. Maternal serum samples collected at 30–35 weeks of gestation and cord blood samples obtained at delivery were analyzed for zonulin and total oxidant status. Associations were examined using both categorical and continuous measures of gestational weight gain.

**Results:**

Higher gestational weight gain was associated with increased maternal and fetal serum zonulin concentrations. Maternal and fetal zonulin levels were positively correlated with each other, and maternal zonulin showed a modest inverse association with neonatal birth weight. Analyses treating gestational weight gain as a continuous variable yielded consistent findings. Given the sample size, results should be interpreted cautiously.

**Conclusions:**

This exploratory study suggests that higher gestational weight gain is associated with increased maternal and fetal serum zonulin concentrations among women with normal pre-pregnancy body mass index. Given the cross-sectional design and modest sample size, causal inferences cannot be drawn. The findings should be considered hypothesis-generating and warrant confirmation in larger, longitudinal studies incorporating comprehensive metabolic and inflammatory assessments.

**Supplementary Information:**

The online version contains supplementary material available at 10.1186/s40795-026-01292-6.

## Introduction

The prevalence of excessive gestational weight gain (GWG) is increasing worldwide, affecting up to 47% of pregnancies [1]. Excessive GWG has been associated with a range of adverse outcomes, including fetal macrosomia [2], unfavorable neurodevelopmental and behavioral consequences [3], and an increased risk of obesity in childhood and adolescence [4], underscoring its clinical significance beyond baseline maternal adiposity.

Growing evidence suggests that maternal metabolic status during pregnancy is closely linked to gut microbiota composition and intestinal barrier function. Maternal overweight and obesity have been associated with reduced gut microbial diversity [5], and alterations in microbiota richness and taxonomic composition have been shown to correlate with serum zonulin concentrations [6, 7]. Serum zonulin (S-zonulin) has been proposed as a marker of intestinal permeability [7], with elevated levels reflecting impaired intestinal barrier integrity [8]. Increased zonulin concentrations have been reported in obesity, obesity-related insulin resistance, and both type 1 and type 2 diabetes [9], whereas healthy body mass index (BMI) and optimal nutritional status have been negatively associated with serum zonulin levels [6].

While previous studies have examined S-zonulin in overweight and obese populations [7] and evaluated zonulin changes following weight-loss interventions [10], data on zonulin dynamics in relation to gestational weight gain within or above recommended ranges are scarce. Gestational weight gain reflects the complex nutritional and metabolic adaptations of pregnancy [7]. Gestational weight gain represents not only energy balance but also the cumulative effects of dietary quality, metabolic regulation, and pregnancy-related nutritional adaptations. Although GWG within recommended limits supports optimal fetal development, excessive weight gain may induce metabolic and inflammatory stress, potentially affecting intestinal barrier function. Clarifying this relationship may improve understanding of the biological pathways linking GWG to adverse pregnancy outcomes.

Evidence regarding maternal–fetal intestinal permeability during pregnancy remains limited, particularly studies that simultaneously assess maternal and fetal biomarkers. Moreover, most existing research has focused either on maternal metabolic markers or neonatal outcomes in isolation and has largely included overweight or obese women, in whom baseline alterations in intestinal permeability are already present. Consequently, the independent contribution of gestational weight gain, separate from pre-pregnancy adiposity, remains unclear.

By restricting the study population to women with normal pre-pregnancy BMI, allows gestational weight gain to be examined as a distinct and clinically relevant exposure, thereby strengthening the novelty of the study. This approach strengthens the novelty of the study and allows a clearer evaluation of the potential association between GWG and maternal–fetal intestinal permeability.

Therefore, this study aimed to explore the association between gestational weight gain and intestinal permeability using serum zonulin concentrations in both maternal and cord blood samples among women with normal pre-pregnancy BMI. The primary outcome of this study was maternal serum zonulin concentration. Secondary outcomes included fetal zonulin levels, total oxidant capacity markers, and neonatal anthropometric measures. Given the observational and cross-sectional design, the study was intended as an exploratory, hypothesis-generating investigation, and the observed associations should not be interpreted as causal. Importantly, dietary intake and gut microbiota composition were not directly assessed; thus, serum zonulin was used solely as a surrogate marker of intestinal permeability without inferring specific dietary or microbiota-mediated mechanisms.

### Study design

This study was designed as a cross-sectional observational study conducted among pregnant women and their neonates. A total of 51 pregnant women in their third trimester (30–35 weeks of gestation) and their neonates were included.

Participants were recruited during pregnancy between 30 and 35 weeks of gestation, and final eligibility was confirmed after delivery based on predefined exclusion criteria, including mode of delivery. Gestational weight gain was evaluated as a clinical exposure variable and categorized according to the recommendations of the U.S. Institute of Medicine (IOM). No direct assessment of dietary intake or energy balance was performed.

### Ethics approval and consent to participate

The study was approved by the Clinical Research Ethics Committee of the Etlik Zübeyde Hanım Women’s Health Training and Research Hospital. All procedures were conducted in accordance with the Declaration of Helsinki. Written informed consent was obtained from all participants prior to enrollment.

Pregnant women aged 18–35 years with a pre-pregnancy body mass index (BMI) within the normal range (18.5–24.9 kg/m²), who were under routine follow-up at the Obstetrics and Gynecology Clinic and who, during the third trimester (30th–35th weeks), had gained weight either within the ranges recommended by the Institute of Medicine (IOM) (11.5–16 kg) or in excess of those recommendations (> 16 kg), were eligible for inclusion.

Exclusion criteria included multiple pregnancy, pre-existing chronic inflammatory or metabolic diseases, gastrointestinal disorders, smoking, use of systemic antibiotics or probiotics during pregnancy, and pregnancy complications such as preeclampsia or gestational diabetes. Women who delivered by caesarean section were excluded to minimize heterogeneity related to obstetric indications and delivery-related factors that could independently influence neonatal outcomes. Potential confounding was partially addressed through strict inclusion and exclusion criteria; however, residual confounding cannot be excluded.

Sample size was calculated based on maternal serum zonulin concentration as the primary outcome using an independent two-sample t-test with a two-sided alpha level of 0.05 and 80% power. Effect size estimates were derived from the study by Demir et al. [11]. The power analysis indicated that a minimum of 19 participants per group (total *n* = 38) was required.

Dietary intake was not assessed using a validated dietary questionnaire or recall method. Given that gestational weight gain reflects a complex interplay of metabolic and behavioral factors during pregnancy, the study focused on the association between gestational weight gain and intestinal permeability rather than specific dietary components.

The English version of the questionnaire used in this study is provided as Supplementary File 1.

### Data collection tools

Demographic and clinical data, including maternal age, pre-pregnancy weight, parity, gestational age, and neonatal birth weight and length, were obtained from the hospital’s electronic medical record system. As participants were enrolled during pregnancy, pre-pregnancy weight was based on maternal self-report at the first prenatal visit rather than direct measurement prior to conception.

Although routine prenatal ultrasonography was performed as part of standard clinical care, ultrasound-based fetal growth parameters and centile classifications were not systematically collected for the purposes of this study.

Maternal weight and height measurements were obtained during routine visits between 30 and 35 weeks of gestation, in accordance with World Health Organization (WHO) guidelines [12, 13]. Body mass index (BMI) was calculated as weight in kilograms divided by height in meters squared and classified according to WHO standards (Geneva, 2000).

Maternal venous blood samples were collected between 30 and 35 weeks of gestation, and umbilical cord blood samples were obtained at delivery. Blood samples were centrifuged within 30 min of collection, and serum aliquots were stored at − 80 °C until analysis. All samples were thawed only once prior to laboratory analysis.

Serum zonulin concentrations were measured using a commercial sandwich enzyme-linked immunosorbent assay (ELISA) kit according to the manufacturer’s instructions (Sandwich-ELISA; Elabscience^®^, Catalog No. EEL-H5560; Wuhan, China). The analytical sensitivity (limit of detection) of the assay was 0.5 ng/mL. The intra-assay and inter-assay coefficients of variation were < 8% and < 10%, respectively, as reported by the manufacturer. Calibration curves were generated using standards provided with the kit.

Total oxidant status (TOS) was determined using a commercially available assay kit (Rel Assay Diagnostics, Gaziantep, Turkey) according to the manufacturer’s instructions and was assessed as a complementary indicator of oxidative stress.

Absorbance values were measured at the appropriate wavelength using a microplate reader (BioTek ELx800, BioTek Instruments, Winooski, VT, USA).

### Statistical analyses

Statistical analyses were performed using SPSS software (version XX.X; IBM Corp., Armonk, NY, USA). Continuous variables were assessed for normality using the Shapiro–Wilk test and visual inspection of histograms. Normally distributed variables are presented as mean ± standard deviation, whereas non-normally distributed variables are presented as median (25th–75th percentile).

Given the exploratory design and limited sample size, analyses were intentionally focused on descriptive statistics and a limited number of inferential comparisons aligned with the primary study objectives.

Between-group comparisons were conducted using independent-samples t-tests for normally distributed variables and the Mann–Whitney U test for non-normally distributed variables. Gestational weight gain was evaluated both as a categorical variable (within vs. above recommended ranges) and as a continuous variable in univariable analyses. To minimize the risk of overfitting, gestational weight gain was not simultaneously included with other correlated covariates in the same multivariable model.

Multiple linear regression analysis was performed with maternal serum zonulin concentration as the dependent variable. Independent variables were selected a priori based on biological plausibility and prior literature, with careful restriction of the number of predictors to maintain an acceptable predictor-to-observation ratio. Multicollinearity was assessed using variance inflation factors.

All statistical tests were two-sided, and a *p* value < 0.05 was considered statistically significant. Given the exploratory nature of the study, no formal adjustment for multiple comparisons was applied to avoid excessive type II error.

## Results

The study comprised 51 women with a mean age of 27.54 ± 5.15 years (range: 18–42 years) and a mean pre-pregnancy BMI of 23.18 ± 2.01 kg/m^2^ (range: 18.61–24.51 kg/m^2^).

Maternal age, pre-pregnancy body mass index, gestational age at delivery, and parity did not differ significantly between women with normal and excessive gestational weight gain (*p* > 0.05). Similarly, neonatal birth weight and birth length were comparable between the two groups (*p* = 0.642 and *p* = 0.680, respectively) (Table 1).

Continuous variables were summarized according to their distribution. Pre-pregnancy BMI showed a normal distribution and was therefore presented as mean ± SD, whereas gestational age, neonatal birth weight, and birth length were not normally distributed and were summarized as median (25th–75th percentile).

**Table 1:** Maternal and neonatal variable by gestational weight gain category

Variable	Normal GWG (*n*:18)	Excessive GWG (*n*:33)	*p*
Maternal age (years), median (25th–75th percentile)	26.3 (23–29)	28.2 (25–32)	0.197
Pre-pregnancy BMI (kg/m²), mean ± SD	21.0 ± 2.3	23.2 ± 1.8	0.112
Gestational age at delivery (weeks), median (25th–75th percentile)	38.2 (37–39)	38.6 (38–40)	0.350
n (%) of those who have given birth to more than one child	13 (72.2%)	26 (78.8%)	0.441
Neonatal birth weight (g), median (25th–75th percentile)	3327 (3100–3600)	3382 (2950–3500)	0.642
Neonatal birth length (cm), median (25th–75th percentile)	50 (49–52)	50 (48–52)	0.680

* Continuous variables are presented as mean ± standard deviation or median (25th–75th percentile), according to data distribution. Pre-pregnancy BMI showed a normal distribution and was compared using the independent-samples t-test. Non-normally distributed continuous variables were compared using the Mann–Whitney U test. Categorical variables are presented as number (percentage) and were compared using the chi-square test or Fisher’s exact test, as appropriate. A *p* value < 0.05 was considered statistically significant

Mean maternal and fetal S-zonulin concentrations were 16.11 ± 8.36 ng/mL and 18.17 ± 11.39 ng/mL, respectively; mean maternal and fetal TOS values were 7.69 ± 7.99 mmol/L and 6.96 ± 9.01 mmol/L, respectively (Table 2).

**Table 2:** Upper and lower means of S-zonulin (ng/mL) and TOS (mmol/L) levels of individuals (n:51)

Variable	Lower and upper values	$\stackrel{-}{\boldsymbol{X}}$±SD
Maternal S-zonulin (ng/mL)	3.87–33.39	16.11 ± 8.36
Fetal S-zonulin (ng/mL)	4.30-51.78	18.17 ± 11.39
Maternal serum TOS (mmol/L)	1.71–47.90	7.69 ± 7.99
Fetal serum TOS (mmol/L)	1.54–61.07	6.96 ± 9.01

**Fig. 1:**
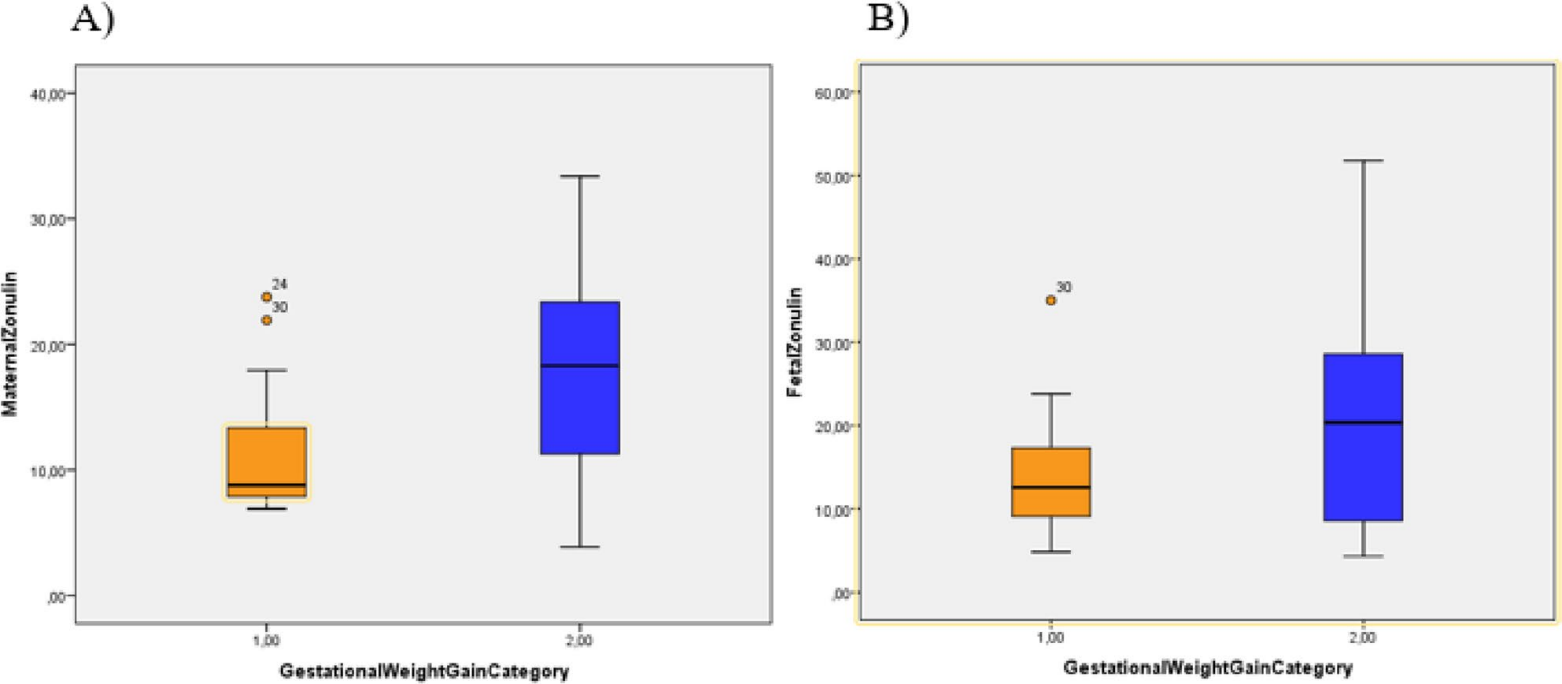
Distribution of maternal (**A**) and fetal (**B**) serum zonulin concentrations according to gestational weight gain category. *Boxplots showing maternal and fetal serum zonulin concentrations according to gestational weight gain category. Colors indicate gestational weight gain category. Boxes represent the 25th–75th percentile, horizontal lines indicate median values, and whiskers indicate minimum and maximum values

Maternal serum zonulin concentrations were significantly higher in women with excessive gestational weight gain compared with those who gained weight within recommended limits (*p* = 0.02). Fetal zonulin concentrations showed a similar pattern (*p* = 0.04). Maternal and fetal TOS levels did not differ significantly between the groups (Table 3; Fig. 1).

**Table 3:** Maternal and fetal serum zonulin and TOS concentrations according to gestational weight gain

Variable	Normal GWG (*n*:18)	Excessive GWG (*n*:33)	*p*
Maternal S-zonulin (ng/mL)	11.5 (3.8–14.8)	18.5 (13.2–33.4)	0.002
Fetal S-zonulin (ng/mL)	14.2 (4.3–18.9)	20.2 (14.5–51.8)	0.044
Maternal serum TOS (mmol/L)	8.0 (1.7–8.6)	7.5 (3.3–47.9)	0.823
Fetal serum TOS (mmol/L), median (25th–75th percentile)	6.1 (1.5–9.8)	7.3 (3.1–61.0)	0.619

* Values are presented as median (25th–75th percentile). Between-group comparisons were performed using the Mann–Whitney U test

Multiple linear regression analysis was performed to identify independent predictors of maternal serum zonulin levels (Table 4).

Fetal serum zonulin levels were positively associated with maternal serum zonulin levels (standardized β = 0.389, *p* = 0.014), whereas neonatal birth weight showed a weak inverse association (standardized β=−0.767, *p* = 0.049). Maternal serum TOS, fetal serum TOS, gestational age at delivery, and neonatal birth length were not significantly associated with maternal serum zonulin levels (*p* > 0.05).

In an additional multivariable model including gestational weight gain (modeled as a continuous variable), maternal age, and gestational age at delivery, gestational weight gain remained independently associated with maternal serum zonulin levels (β = 0.29, 95% CI: 0.05–0.53, *p* = 0.02). No substantial multicollinearity was observed among the predictors included in the final models.

**Table 4:** Multiple linear regression analysis of maternal serum zonulin levels

Variable	Standardized β	T	*p*
Maternal serum TOS (mmol/L)	0.037	0.231	0.819
Fetal serum zonulin (ng/mL)	0.389	2.572	0.014*
Fetal serum TOS (mmol/L)	0.014	0.090	0.929
Birth weight (g)	−0.767	−1.711	0.049*
Birth length (cm)	0.911	2.035	0.095
Gestational age (weeks)	−0.004	−0.026	0.980

*Standardized regression coefficients (β) are presented, * *p* < 0.05,. Effect sizes for the regression analyses are presented as standardized regression coefficients (β) to facilitate interpretation of the magnitude and direction of associations

Gestational weight gain remained independently associated with maternal serum zonulin levels (β = 0.29, 95% CI: 0.05–0.53, *p* = 0.02). No substantial multicollinearity was observed among the predictors included in the final model.

## Discussion

In this study of women with normal pre-pregnancy BMI, excessive gestational weight gain was associated with higher maternal and fetal serum zonulin concentrations, and maternal zonulin levels were inversely associated with neonatal birth weight.

By focusing on women with normal pre-pregnancy BMI, the present study extends existing literature by examining gestational weight gain as a relatively independent exposure, thereby minimizing potential confounding by pre-existing overweight or obesity.

This pattern warrants careful interpretation, as it contrasts with the generally expected positive association between gestational weight gain and fetal growth. Although excessive gestational weight gain is typically associated with increased fetal growth, the inverse association observed between maternal zonulin levels and neonatal birth weight may reflect complex physiological processes that were not directly measured in this study. Given the cross-sectional design, the directionality and causality of this relationship cannot be determined.

Antibiotic therapy during birth has been shown to increase neonatal stool zonulin concentrations on day 7, with similarly elevated umbilical cord serum concentrations observed when antibiotics were administered during pregnancy [14]. Pregnant women receiving antibiotic therapy during pregnancy were excluded from the present study, thereby reducing the likelihood of this potential confounding factor.

During pregnancy, maternal physiological adaptations support fetal growth and development. Gestational weight gain and fetal growth are influenced by multiple interacting factors, including genetic, metabolic, inflammatory, and placental determinants [15]; however, these factors were not directly assessed in the present study.

Data on zonulin concentrations in the neonatal period remain limited. Tarko et al. [16] compared zonulin concentrations measured by ELISA in neonatal patients with various clinical conditions, including sepsis, extremely low birth weight, necrotizing enterocolitis, rotavirus infection, and abdominal wall defects, and reported significantly higher zonulin levels in neonates with rotavirus infection and abdominal wall defects compared with controls. Similarly, Saleem et al. [17] observed lower zonulin concentrations in neonates born before 28 weeks of gestation than in term controls. Although these studies were conducted in postnatal and clinically heterogeneous neonatal populations, they indicate that zonulin concentrations may vary according to developmental stage and clinical condition. Within this context, the concurrent assessment of maternal and fetal zonulin concentrations at birth in the present study adds complementary data to the existing literature.

Although serum zonulin was used as a marker of intestinal permeability in the present study, the observed inverse association with fetal growth may reflect complex maternal–fetal physiological processes that were not directly assessed, rather than direct effects of intestinal nutrient absorption.

As placental structure, perfusion, and nutrient transport were not evaluated, the relative contribution of intestinal versus placental pathways cannot be determined. It is also possible that increased zonulin levels reflect a secondary association or epiphenomenon related to altered fetal growth rather than a causal factor.

Serum zonulin has been proposed as a surrogate marker of intestinal permeability, although its interpretation remains subject to ongoing discussion [18]. Elevated zonulin levels have been associated with impaired intestinal barrier function and metabolic disturbances in some populations [19]; however, inflammatory and metabolic markers were not assessed in the present study. Therefore, any interpretation regarding inflammation should be considered cautious.

Previous studies have reported elevated zonulin levels in obese and metabolically compromised populations; however, evidence during pregnancy remains limited. Experimental studies suggest a relationship between intestinal permeability and obesity-related metabolic changes [20–22], and zonulin has been shown to modulate paracellular tight junctions, potentially influencing intestinal barrier function [23]. Nevertheless, the clinical and mechanistic implications of these findings during pregnancy remain uncertain.

Studies in obese individuals and patient populations have reported positive correlations between zonulin concentrations, intestinal permeability, and inflammatory cytokines [7, 24]. However, inflammatory markers were not assessed in the present study, and therefore direct comparisons should be made cautiously. Within this context, the concurrent assessment of maternal and fetal zonulin concentrations during pregnancy adds to the limited body of literature in this area.

Reduced intestinal permeability has been reported following weight loss in individuals with severe obesity [10], whereas increased zonulin levels have been observed after dietary interventions in individuals with type 2 diabetes (mean BMI ~ 30 kg/m²) [16]. While several longitudinal studies have evaluated zonulin dynamics before and after weight loss interventions [10, 25], data specifically examining changes in relation to gestational weight gain remain limited.

The observed increase in maternal and fetal zonulin levels in association with greater gestational weight gain may reflect broader physiological adaptations during pregnancy rather than specific dietary factors. However, metabolic and inflammatory parameters were not directly assessed, and therefore mechanistic interpretations should be made cautiously. An important strength of the present study is the concurrent assessment of maternal and fetal serum zonulin concentrations, an area for which data during pregnancy remain limited.

## Conclusion

In conclusion, this study provides preliminary, hypothesis-generating evidence of an association between gestational weight gain and maternal–fetal zonulin concentrations among women with normal pre-pregnancy BMI. Given the cross-sectional design and the use of zonulin as a surrogate marker of intestinal permeability, these findings should be interpreted cautiously and warrant confirmation in larger, longitudinal studies incorporating more comprehensive physiological and metabolic assessments.

This study have several limitations. Recent evidence has raised concerns regarding the specificity of commercial ELISA kits for zonulin measurement, as some assays may detect structurally related proteins (e.g., properdin or pre-haptoglobin 2) rather than zonulin itself [26, 27]. Therefore, serum zonulin should be interpreted cautiously as a proxy marker of intestinal permeability rather than a direct quantification of zonulin activity. Inflammatory biomarkers, maternal dietary intake, physical activity, socioeconomic factors, and fecal microbiome composition were not assessed in this study. Given the well-documented associations among gut microbiota, inflammation, metabolic regulation, and intestinal permeability, the absence of these measurements limits mechanistic interpretation of the observed associations. Accordingly, any proposed biological pathways should be considered as hypothetical. Serum zonulin was used solely as a functional surrogate marker of intestinal permeability, and direct microbiome assessment was not feasible within the framework of this exploratory study.

Unequal group sizes reflected the natural distribution of gestational weight gain in the study population; however, this imbalance may have reduced statistical power and limited the precision of between-group comparisons. Although the final sample size exceeded the minimum requirement estimated in the power analysis, unequal group distribution should be considered when interpreting the findings.

Pre-pregnancy weight was self-reported and not directly measured prior to conception, which may have introduced recall bias and potential misclassification of pre-pregnancy BMI. Fetal growth was assessed only at birth using anthropometric measurements, and ultrasound-based growth trajectories or birthweight centiles were not available.

Dietary intake and energy balance were not assessed; accordingly, gestational weight gain should not be interpreted as a proxy for nutritional composition.

The absence of correction for multiple testing may increase the risk of type I error; therefore, the findings should be regarded as hypothesis-generating rather than confirmatory. Larger, longitudinal studies are required to confirm these associations and to clarify their physiological and clinical relevance.

Given the cross-sectional observational design, all findings should be interpreted as associations rather than causal relationships. Future longitudinal and interventional studies are warranted to clarify the potential clinical implications of intestinal permeability markers during pregnancy.

## Supplementary Information

Supplementary Material 1.

## Data Availability

The datasets used and/or analysed during the current study are available from the corresponding author on reasonable request.

## Abbreviations

GWG: Gestational weight gain
S-zonulin: Serum zonulin
BMI: Body mass index
IOM: Institute of Medicine
TOS: Total Oxidant Capacity
