# Supplementary material for: Maternal weight gain and intestinal permeability marker zonulin in late pregnancy: implications for nutritional and metabolic health
Source: BMC Nutr. 2026 Feb 27;12:63. doi: 10.1186/s40795-026-01292-6 (PMC13049713; doi:10.1186/s40795-026-01292-6)
Supplement: Supplementary file 1 — Supplementary Material 1. [file 40795_2026_1292_MOESM1_ESM.doc]

**Evaluation Of The Effect Of Body Weight Gain During Pregnancy On Maternal Serum and Neonal Cord Blood Zonulin Levels**

**Patient Follow-Up Form**

**Age (year) :……………………………**

**Number of pregnancies :……………………………**

**Pre-pregnancy body weight :……………………………(kg)**

**Height :……………………………(cm)**

**Body weight (3rd trimester) :……………………………(kg)**

**Newborn birth weight : ……………………………(gr)**

**Newborn birth size : ……………………………(cm)**

**Week of gestation : ……………………………**
